# Apolipoprotein Eε4: A Biomarker for Executive Dysfunction among Parkinson's Disease Patients with Mild Cognitive Impairment

**DOI:** 10.3389/fnins.2017.00712

**Published:** 2017-12-20

**Authors:** Nor A. Samat, Nor A. Abdul Murad, Khairiyah Mohamad, Mohd R. Abdul Razak, Norlinah Mohamed Ibrahim

**Affiliations:** ^1^Department of Medicine, UKM Medical Centre, Chancellor Tuanku Muhriz Hospital & Universiti Kebangsaan Malaysia Medical Centre, Kuala Lumpur, Malaysia; ^2^Universiti Kebangsaan Malaysia Medical Molecular Biology Institute, Kuala Lumpur, Malaysia

**Keywords:** Parkinson's disease, mild cognitive impairment, Apolipoprotein E, alpha synuclein, MoCA, PDCRS, CTMT

## Abstract

**Background:** Cognitive impairment is prevalent in Parkinson's disease (PD), affecting 15–20% of patients at diagnosis. α-synuclein expression and genetic polymorphisms of Apolipoprotein E (*ApoE*) have been associated with the presence of cognitive impairment in PD although data have been inconsistent.

**Objectives:** To determine the prevalence of cognitive impairment in patients with PD using Montreal Cognitive Assessment (MoCA), Comprehensive Trail Making Test (CTMT) and Parkinson's disease-cognitive rating scale (PDCRS), and its association with plasma α-synuclein and *ApoE* genetic polymorphisms.

**Methods:** This was across-sectional study involving 46 PD patients. Patients were evaluated using Montreal cognitive assessment test (MoCA), and detailed neuropsychological tests. The Parkinson's disease cognitive rating scale (PDCRS) was used for cognitive function and comprehensive trail making test (CTMT) for executive function. Blood was drawn for plasma α-synuclein measurements and *ApoE* genetic analysis. *ApoE* polymorphism was detected using MutaGEL*APoE* from ImmunDiagnostik. Plasma α-synuclein was detected using the ELISA Technique (USCN Life Science Inc.) according to the standard protocol.

**Results:** Based on MoCA, 26 (56.5%) patients had mild cognitive impairment (PD-MCI) and 20 (43.5%) had normal cognition (PD-NC). Based on the PDCRS, 18 (39.1%) had normal cognition (PDCRS-NC), 17 (37%) had mild cognitive impairment (PDCRS-MCI), and 11 (23.9%) had dementia (PDCRS-PDD). In the PDCRS-MCI group, 5 (25%) patients were from PD-NC group and all PDCRS-PDD patients were from PD-MCI group. CTMT scores were significantly different between patients with MCI and normal cognition on MoCA (*p* = 0.003). Twenty one patients (72.4%) with executive dysfunction were from the PD-MCI group; 17 (77.3%) with severe executive dysfunction and 4 (57.1%) had mild to moderate executive dysfunction. There were no differences in the plasma α-synuclein concentration between the presence or types of cognitive impairment based on MoCA, PDCRS, and CTMT. The*ApoEe4* allele carrier frequency was significantly higher in patients with executive dysfunction (*p* = 0.014).

**Conclusion:** MCI was prevalent in our PD population. PDCRS appeared to be more discriminatory in detecting MCI and PDD than MoCA. Plasma α-synuclein level was not associated with presence nor type of cognitive impairment, but the *ApoEe4* allele carrier status was significantly associated with executive dysfunction in PD.

## Introduction

Parkinson's disease (PD) is the second commonest neurodegenerative disease after Alzheimer's disease with increased prevalence after the age of 40 (Pringsheim et al., 2014). It affects 1% of individuals older than 60 years (Schrag and Schott, 2006). The diagnosis of PD is purely clinical, relying mainly on physical findings of bradykinesia and akinesia, tremor, rigidity, and also postural instability (Nutt and Wooten, 2005). In addition to motor symptomatology, PD is now recognized to be a complex disorder with diverse clinical features which includes non-motor manifestations (NMS) (Palavra et al., 2013). These NMS are often poorly recognized and inadequately treated; hence contributing to significant long term disability, impaired quality of life, and reduced life expectancy (Hughes et al., 2004). Cognitive impairment, a neuropsychiatric NMS, has a prevalence of 28.6% at 5 years of disease, predominating in later stages of PD (Riedel et al., 2008). Cognitive impairment in PD comprises of a broad spectrum of clinical deficits and severity, affecting both the non-amnestic and amnestic domains (Leverenz et al., 2009). Pathogenesis of this impairment has been frequently attributed to neurochemical alterations in the dopaminergic, cholinergic, and other neurotransmitter systems, and to neuro-pathological contributions of limbic and cortical Lewy bodies and neurites, amyloid deposition, neurofibrillary tangles, and cerebrovascular disease (Leverenz et al., 2009). Executive dysfunction reflects impairment in the frontal lobes, particularly the dorsolateral prefrontal cortex, resulting from disruptions of fronto-striatal loops, due to degeneration of either the dopaminergicnigrostriatal or mesocortical pathways (Goldman and Litvan, 2011). The presence of mild cognitive impairment is frequently missed as screening test for cognition is not routinely performed in the outpatients setting, unless patients or relatives complain of memory difficulties. Mild cognitive impairment (MCI) is now recognized to be a risk factor for developing PD dementia (Janvin et al., 2003). Recognizing and identifying MCI early in the disease, will allow for better treatment and follow-up strategies to minimize the risk of developing overt dementia.

The underlying pathogenesis of cognitive impairment in PD is complex and involves the interaction between the environmental and genetic factors (Morley et al., 2012). Recent literature suggest that *ApoE* polymorphism may alter the risk of dementia in PD patients (Huang et al., 2004, 2006; Williams-Gray et al., 2009). There are 6 genotypes and *ApoEe3/e3* is the most common genotype (Eichner et al., 2002). An earlier study evaluating the role of *ApoE* in dementia associated with PD was inconclusive (Koller et al., 1995). *ApoEe4* has been later shown to be associated with sporadic PD (Huang et al., 2004) and with dementia in PD (Huang et al., 2006). Another study reported that carrying at least one *ApoEe4* allele was associated with a more rapid cognitive decline in PD (Morley et al., 2012). α*-synuclein* also is known to be associated with PD (Simon-Sanchez et al., 2009). However, it is unclear how alterations of the *SNCA* gene cause Parkinson disease. More recently, plasma and CSFα*-synuclein* has been shown to be associated with cognitive decline in PD (Emamzadeh, 2017) and as a potential biomarker of cognitive decline in PD.

As PD patients may have early cognitive impairment even without overt memory complaints, this study was therefore conducted to determine the prevalence of mild cognitive impairment among PD patients using MoCA and PDCRS (Level 1 Movement Disorders Criteria) (Litvan et al., 2012). We also determined the prevalence of executive dysfunction using the CTMT and correlated the presence of cognitive impairment with measurable biomarkers such as *ApoE*4 and plasma α-synuclein.

## Materials and methods

### Study design and selection of population

This was a cross sectional study conducted in Universiti Kebangsaan Malaysia Medical Centre (UKMMC) from March to September 2013. The study was approved by the UKMMC Ethics & Research Committee. All patients had provided written informed consent to participate in this study. The subjects were recruited from the neurology outpatient clinic in PPUKM. They were selected based on the standard inclusion and exclusion criteria. Inclusion criteria: Patients with the diagnosis of PD by a neurologist (UK PD Society Brain Bank Criteria) (Hughes et al., 1992) and patients who scored ≥18 on Montreal Cognitive Assessment (MoCA). Exclusion criteria: Patients with coexisting Alzheimer's disease or vascular dementia; idiopathic PD with Hoehn & Yahr stage 4–5; patients with ischemic heart disease or atherosclerosis; patients with type 111 hyperlipoproteinaemia or familial dysbetalipoproteinaemia.

#### Assessments

Clinical assessments, including the relevant demographic data levels of education were recorded. Educational level was categorized into primary (6 years of education), secondary (11–13 years of education) and tertiary (14 years and onwards). MoCA test was performed by AS. PDCRS and CTMT were performed in all patients by a trained neuropsychologist KM. Parkinson's Disease was staged using the Hoehn & Yahr classification. The cognitive assessment was performed in the ON state, to ensure full cooperation of the patients and to reduce any confounding effects of OFF symptoms on cognition.

### Cognitive tests

#### Montreal cognitive assessment (MoCA)

Montreal Cognitive Assessment (MoCA) is a cognitive screening instrument that was designed to address some of the limitations of the MMSE (Nasreddine et al., 2005). Based on the MoCA scores, the patients were further categorized as normal cognition (PD-NC) if their MoCA scores were 26–30 or mild cognitive impairment (PD-MCI) if their scores were 18–25 based on a Malaysian study (Normah et al., 2016). PD patients who scored less than 18 were excluded, as we wanted to include only those with normal or mild cognitive impairment.

#### Parkinson's disease cognitive rating scale (PDCRS)

All patients were subjected to PDCRS. PDCRS is designed to cover the full spectrum of cognitive defects associated with PD. It includes items that assess fronto-subcortical defects and cortical dysfunction (Pagonabarraga et al., 2008). Based on the PDCRS scores, patients were the categorized into those with normal cognition (PDCRS-NC; score 82–134) (Fernández de Bobadilla et al., 2013) and dementia (PDCRS-dementia; score 0–64) (Pagonabarraga et al., 2008). Based on these two papers, the PDCRMS MCI scores were obtained (65–81).

#### Comprehensive trail making test (CTMT)

All patients were subjected to CTMT. This test comprises a standardized set of five visual search and sequencing tasks that are heavily influenced by attention, concentration, resistance to distraction, and cognitive flexibility (or set-shifting), in addition to the more obvious visual search and sequencing demands of the tasks. This test is a sensitive measures of executive function (Stuss et al., 2001). It is useful in assessing brain damage or dysfunction of dementia, detection of attention and concentration deficits, and frontal activation defects. From the test, patients were categorized into those with executive and without executive dysfunction. Further categorization into severely impaired, mild to moderately impaired, low average and average were done according to the CTMT manual. The qualitative descriptions of performance levels were expressed as T-scores for the CTMT trails and CTMT Composite Index.

### Detection of apolipoprotein-E polymorphism

*ApoE* polymorphism was detected using MutaGEL*APoE* from ImmunDiagnostik, Germany. Polymerase chain reaction (PCR) for codons 112 and 158 of the *ApoE* gene was performed according to the protocol described by the manufacturer. The PCR products were digested using the restriction enzymes *ApoE* 1 and 2. Electrophoresis was performed to view the restriction digestion products and results were interpreted as described in the protocol (MutaGel®*ApoE*, ImmunDiagnostik, Germany). PCR reaction was prepared in a 0.2 μL microtube where 5 μL of the sample was added to 20 μL of the PCR mix. Positive and negative controls (distilled water) were also included in separate reactions. PCR was performed following these steps, initial hold at 95°C for 5 min, followed by 38 cycles of 95°C for 30 s/62°C for 30 s/72°C for 60 s, final hold at 72°C for 5 min in My Cycler (Biorad Laboratories, USA). PCR products were digested according to the protocol. Gel electrophoresis was carried out in more than 3.5% agarose for at least 150 Vh in 1 × TBE-buffer. The separated DNA was visualized under UV-light (312 nm) in a gel documentation system (BioRad, USA). The presence of *ApoE* carrier was based on the PCR fragments:191 ± 142 + 49/118 ± 93 + 25 was for*ApoE*2/*ApoE*4, 191 ± 142 + 49/93 + 25 for *ApoE*3/*ApoE*4, 191/93 + 25 for *ApoE*4/*ApoE*4 and 212 ± 191/93 + 25 for Leiden/*Apo E4*. PCR bands should appear at underlined numbers for each of the genotypes. Apo E Leiden is a rare variant of human ApoE.

### Plasma α-synuclein quantification using elisa

Plasma α-synuclein was detected using the enzyme-linked immuno assay technique (ELISA) (USCN Life Science Inc., USA) according to standard protocol. Briefly, the stock for standard (2,000 pg/mL) was diluted to 1,000, 500, 250, 125, 62.5, 31.2, 15.6 pg/mL with Standard Diluent. Blank was prepared using Standard Diluent alone at 0 pg/mL. The detection Reagent A and B were diluted with Assay Diluent A and B, respectively at 1 in 100 × and to the working concentration of 2,000, 1,000, 500, 250, 125, 62.5, 31.2, 15.6, and 0 pg/mL. The Wash Solution concentrate (30 ×) was diluted with 580 mL of deionized or distilled water to prepare 600 mL of Wash Solution (1 ×). About 20 μL plasma sample was used and diluted with 180 μL PBS. In total, 7 wells were prepared for standard and 1 well for blank. 100 μL of dilutions of standard, blank and samples were added into the appropriate wells. The plate was covered with the plate sealer and incubated for 2 h at 37°C. The liquid was removed and 100 μL of Detection Reagent A working solution was added to each well. The reaction was incubated for 1 h at 37°C after covering it with the Plate sealer. The reagents were aspirated and washed with 350 μL of 1 × Wash Solution to each well. The wells were air dried for 2 min. The wells were washed 3 times and 100 μL of Detection Reagent B working solution was added to each well. The plate was incubated for 30 min at 37°C. Washed 3 times and 90 μL of Substrate Solution was added to each. The plate was incubated at 37°C for 15–25 min and must be protected from light. About 50 μL of Stop Solution was added to each well and removed any water from the plate. The color change was measured spectrophotometrically at a wavelength of 450 ± 10 nm. The concentration of α-synuclein in the samples was then determined by comparing the O.D. of the samples to the standard curve.

### Statistical analysis

Data were entered and analyzed using the SPSS version 21.0. For descriptive analyses, categorical data was described with frequency and percentage (%). For normally distributed continuous data, mean [standard deviation (sd)] was used. Median [interquartil range (IQR) was used for non-normally distributed data. Bivariable analysis for categorical data was conducted using Fisher Exact tests. For continuous data, Student's *t*-test was used for normally distributed data. For non-normally distributed data, Kruskal Wallis and Mann Whitney tests were used. A *p*-value < 0.05 was considered as statistically significant. To minimize any misinterpretation of predictor exclusion (as predictors may be highly correlated with the outcome), the criteria for inclusion and exclusion of a variable was set at a level of α ≤ 0.05 and ≥0.15, respectively. Furthermore, stepwise selection of predictors was specified where predictors were removed from the equation one at a time, but could be retained if doing so would help prediction.

## Results

### Baseline data of recruited patient

Following the screening of 200 patients, only 46 were eligible and recruited into the study based on the inclusion and exclusion criteria. Of these, 29 (63%) patients were male and 17 (37%) were female. Majority of patients were in stage 2 PD (*n* = 26) and the least were in stage 3 (*n* = 5). The majority patients were Chinese (*n* = 26) and the least were Indian (*n* = 2). The median age of study population was 64 (58–67) years, whereas the age of onset of PD was 59 (53–63) years. The median duration of disease was 4 (3–7) years. From the MoCA test, 26 (56.5%) patients had mild cognitive impairment and 20 (43.5%) patients had normal cognitive function.

### Cognitive dysfunction using MoCA

Demographic data for PD-NC and PD-MCI group of patients were presented in Table 1. There were no significant differences in the median age, onset age of disease and disease duration between both groups. There was significant difference in the level of educational between both groups (*p* = 0.025). Only 1 patient (14.3%) in the PD-MCI group received tertiary education while 6 patients (85.7%) in the PD-NC group had tertiary education. 12 patients (41.4%) in the PD-NC group and 17 patients (58.6%) in the PD-MCI group had secondary education. The median age onset of PD was 60 years (54–62) in PD-NC and 58 years (52–63) in PD-MCI groups. Majority of the patients were in stage 2 by Hoehn and Yahr classification; 11 (42.3%) of the PD-NC group and 15 (57.7%) of the PD-MCI group. All five stage 3 patients were in the PD-MCI group. However, the distribution of Hoehn & Yahr stages was not significantly different between PD-NC and PD-MCI. There was a significant difference in the total levodopa equivalent dosage (LED) between the 2 groups with a median value 225 mg per day (115.6–300.0) in the PD-NC group compared to 409.5 mg per day (200.0–707.6) in PD-MCI group (*p* = 0.006). For anticholinergic drugs, 17 (60.7%) of the PD-MCI group and 11 (39.3%) from PD-NC patients were on this medication but this didn't meet statistical significance.

**Table 1:** Demographic and clinical data of PD-NC and PD-MCI patients.

**Demographic data**	**PD-NC *n*(%)**	**PD-MCI *n*(%)**	***p*-value**
Age (years)	64 (58–65)	63 (58–69)	0.748^μ^
**GENDER (%)**			
Male	13 (44.8)	16 (55.2)	0.809^χ^
Female	7 (41.2)	10 (55.8)	
**ETHNICITY (%)**			
Malay	9 (50)	9 (50)	0.736^χ^
Chinese	10 (38.5)	16 (61.5)	
Indian	1 (50)	1 (50)	
**EDUCATION LEVEL (%)**			
Primary	2 (20)	8 (80)	0.025^χ^
Secondary	12 (41.4)	17 (58.6)	
Tertiary	6 (85.7)	1 (14.3)	
**CLINICAL FEATURES**			
Age onset of PD (years)	60 (54–62)	58 (52–63)	0.929^μ^
Duration of PD (years)	4 (2–6)	5 (3–8)	0.523^μ^
**STAGE OF DISEASE (%)**			
Stage 1	9 (60)	6 (40)	0.063^χ^
Stage 2	11 (42.3)	15 (57.7)	
Stage 3	0 (0)	5 (100)	
Total daily levodopa dose (mg/day)	225 (115.6–300.0)	409.5 (200.0–707.8)	0.006^μ^
**ANTICHOLINERGIC (%)**			
Yes	11 (39.3)	17 (60.7)	0.474^χ^
No	9 (50.0)	9 (50.0)	

*For demographic data: Data are presented in either counts (percentage) or median with interquartile range*.

^μ^ *Mann-Whitney U-test, ^χ^Fisher-Exact test*.

*For clinical data: Data are presented in counts (percentage) or median with interquartile range*.

^μ^ *Mann-Whitney U-test, ^χ^Fisher Exact test*.

*Hoehn and Yahr staging for Parkinson's disease*.

### Cognitive dysfunction using PDCRS and CTMT

The PDCRS and CTMT scores were compared between the PD-NC and PD-MCI and presented in Table 2. The PDCRS total score was significantly different between both groups. The mean score was 90 (±11) in the PD-NC group and 68 (±11) in the PD-MCI group (*p* < 0.001). There were also significant differences in the PDCRS subcortical and cortical scores between the two groups. Based on the PDCRS total score, patients were further subdivided into 3 groups; normal cognition (PDCRS-NC), mild cognitive impairment or predementia (PDCRS-MCI) and dementia (PDCRS-PDD). 15 patients (75%) in PD-NC group had normal cognition compared to only 3 patients (11.5%) in PD-MCI. Comparing findings on MoCA with PDCRS, 5 (25%) patients with normal cognition on MoCA had mild cognitive impairment on the PDCRS. In contrast, among the PD-MCI group, 12 patients (46.1%) were still classified as mild cognitive impairment, while 11 patients (42.3%) fulfilled the criteria for dementia in the PDCRS. All these findings were statistically significant with *p*-value of 0.001. Using bivariate analysis, there was a positive significant correlation between MoCA and PDCRS scores (*p* = 0.01).

**Table 2:** Comparison of PDCRS and CTMT in PD-NC and PD-MCI patients.

**PDCRS**	**PD-NC (*n* = 20)**	**PD-MCI (*n* = 26)**	***p*-value**
PD-CRS total score	90 ± 11	68 ± 12	<0.001^θ^
PD-CRS subcortical score	62 ± 10	42 ± 10	<0.001^θ^
PD-CRS cortical score	29 ± 1	26 ± 4	0.002^θ^
**PD-CRS CLASSIFICATION (%)**			
PDCRS-NC	15 (75.0)	3 (11.5)	<0.001^χ^
PDCRS-MCI	5 (25.0)	12 (46.1)	
PDCRS-PDD	0 (0)	11 (42.3)	
CTMT t-score sum	204 (154–226)	137 (100–179)	0.003^μ^
**CTMT CATEGORY**			
With executive dysfunction	8 (27.6)	21 (72.4)	0.005^χ^
Without executive dysfunction	12 (70.6)	5 (29.4)	

*For PDCRS:Data are presented either in n(%) or mean (sd*).

*PDCRS-NC score: 82–134, PDCRS-MCI score: 65–81, PDCRS-PDD score: 0–64*.

^θ^ *Student's t-test, ^χ^Fisher Exact test*.

*For CTMT: Data are presented inn (%) or median (IQR*).

^μ^ *Mann-Whitney U-test, ^χ^Fisher Exact test*.

*Executive dysfunction = severely impaired + mild to moderately impaired*.

*Based on CTMT composite index-severely impaired: < 30, mild to moderately impaired: 30–35, low average: 36–42, average:43–57, high average: 58–64, superior: 65–70, very superior: >70*.

The scores for the CTMT were also significantly different between PD-NC and PD-MCI groups, with scores of 204 (154–226) and 137 (100–179) respectively (*p* = 0.003). Twenty one patients (72.4%) from the PD-MCI group had executive dysfunction. Of these, 17 (77.3%) had severe executive dysfunction and 4 (57.1%) had mild to moderate executive dysfunction. In contrast, only 8 patients (27.6%) from PD-NC group had executive dysfunction and 5 patients (22.7%) had severe executive dysfunction.

### Plasma α-synuclein level and PD cognition

Data on plasma α-synuclein level was available for 40 patients (PD-NC; *n* = 16, PD-MCI; *n* = 24) and tabulated according to the cognitive tests used (Table 3). α-synuclein level was not tested in 6 patients due to lack of plasma sample. The median α-synuclein level for all patients was 18.10 pg/ml (14.42–23.64 pg/ml). There were no significant differences in the α-synuclein level between the PD-NC and PD_MCI groups, with levels of 17.40 pg/ml (13.79 to 19.78 pg/ml) vs. 19.66 pg/ml (15.53 to 25.82 pg/ml) respectively. Similarly, there were also no differences in the level of α-synuclein between the PDCRS groups and CTMT groups as shown in Table 3.

**Table 3:** Levels of α-synuclein according to MoCA, PDCRS and CTMT (*n* = 40).

**Levels**	**MoCA**				***p*-value**
	**PD-NC (*n* = 16)**	**PD-MCI (*n* = 24)**			
Concentration (pg/ml)	17.41 (13.79–19.78)	19.66 (15.53–25.82)			0.151^μ^
	**PDCRS**				
	**PDCRS-NC (*n* = 15)**	**PDCRS-MCI (*n* = 15)**	**PDCRS-PDD (*n* = 10)**		
Concentration (pg/ml)	17.40 (13.96–18.82)	20.61 (15.27–26.27)	19.66 (16.28–30.60)		0.160^k^
	**CTMT**				
	**Executive dysfunction (*n* = 26)**	**No executive dysfunction (*n* = 14)**			
Concentration (pg/ml)	18.10 (14.86–21.69)	18.12 (14.09–26.27)			0.955^μ^
	**CTMT by categories**				
	**Severely impaired (*n* = 19)**	**Mild to moderately impaired (*n* = 7)**	**Low average (*n* = 6)**	**Average (*n* = 8)**	
Concentration (pg/ml)	19.64 (15.30–24.15)	17.49 (13.53–18.10)	15.17 (13.88–21.33)	20.47 (15.81–28.46)	0.534^k^

μ *Mann-Whitney U-test*.

k *Kruskal Wallis test*.

### *ApoE* genetic polymorphism and PD cognition

Data for *e4* allele carriers was tabulated in Table 4. All patients (*n* = 46) were tested for *ApoE* genetic polymorphisms. However, 4 patients had insufficient data for interpretation, due to problems with DNA quality. There were no significant differences in the *ApoE* genetic polymorphisms, particularly the *e4* allele frequency, among the PD-NC and PD-MCI groups. Similarly, there were no significant differences in the *e4* allele frequency in the PDCRS groups. However, there was a significant difference in the *e4* allele frequency between patients with executive dysfunction and without executive dysfunction using the CTMT (*p* = 0.014).

**Table 4:** Distribution of *ApoE* genotypes and *e4* alleles based on MoCA and PDCRS.

**Alleles**	**No *e4***	***e4* present**	**Undetected^a^**	***p*-value**
**MOCA**				
PD-NC	14 (51.9)	5 (33.3)	1 (25)	0.248^χ^
PD-MCI	13 (48.1)	10 (66.7)	3 (75)	
**PDCRS**				
PD-NC	13 (48.1)	5 (33.3)	0 (0)	0.501^χ^
PD-MCI	9 (33.3)	5 (33.3)	3 (75)	
PDD	5 (18.5)	5 (33.3)	1 (25)	
**CTMT**				
Executive dysfunction	13 (48.1)	13 (86.7)	3 (75)	0.014^χ^
No executive dysfunction	14 (51.9)	2 (13.3)	1 (25)	
Severity of executive dysfunction based on CTMT				
Severely impaired	10 (37)	10 (66.7)	2 (50)	0.075^χ^
Mild to moderately impaired	3 (11.1)	3 (20.0)	1 (25)	
Low average	7 (25.9)	2 (13.3)	0 (0)	
Average	7 (25.9)	0 (0)	1 (25)	

*For MoCA:Data are presented in n(%*).

^χ^ *Fisher Exact, ^a^Undetected*.

*For PDRS:Data are presented in n(%*).

^χ^ *Fisher Exact, ^a^Undetected*.

*For executive dysfunction using CTMT: ^χ^Fisher Exact, ^a^Undetected*.

*For severity of executive dysfunction based on CTMT: Data are presented in n(%*).

^χ^ *Fisher Exact, ^a^Undetected*.

## Discussion

In this study, 56% of the PD patients fulfilled the criteria for MCI based on MoCA, with a median duration of disease of 4 years and median age of 64 years. This is somewhat consistent with findings from another study, which found 53.8% out of their 290 PD patients met the criteria for MCI with a mean duration of disease of 9 years and mean age of 68.8 years (Monastero et al., 2012). Another study found that 42/76 (55%) PD patients had MCI with mean age of 73 (Janvin et al., 2003). When compared with our studies, interestingly, MCI was detected in half of our patients, despite a relatively short disease duration. The use of MoCA test, in our study could have increased the detection of MCI, as the other studies used the Mini Mental State examination (MMSE). We also found that our patients with MCI had significantly higher LED requirement, implying that MCI is associated with more severe disease. Anticholinergic drugs are known to cause cognitive impairment in the elderly and also in PD patients (Cooper et al., 1992; Ancelin et al., 2006). However, our study did not show significant difference in the use of anticholinergic medications in patients with MCI and normal cognition, possibly due to the small sample size.

We attempted to correlate the findings from MoCA with more comprehensive neuropsychological test, such as the PDCRS to ascertain the Level 1 MDS criteria of MCI and to further evaluate executive dysfunction using the CTMT. PDCRS, was used for its reliability and validity in accurately diagnosing PDD and detect subtle fronto-subcortical deficits (Pagonabarraga et al., 2008). Based on the PDCRS, only 36.9% of our patients were in the MCI category, 39.2% had normal cognition and surprisingly the remaining 23.9% had been labeled as PDD. This is because 11 out of 26 patients with MCI on MoCA were reclassified as PDD using the PDCRS. In addition, 5 out of 20 patients with normal cognition on MoCA were re-categorized as MCI using the PDCRS. This is perhaps not surprising as PDCRS is more specific for PD, compared to MoCA and thus may have higher sensitivity in detecting MCI and PDD amongst PD patients. Surprisingly, 3/26 patients with MCI on MoCA were classified as normal cognition on the PDCRS, giving a false positive value for MoCA of 11.5%. Our findings are consistent with a previous study involving 234 PD patients in an outpatient setting, which showed that the PDCRS was better at identifying PD-MCI and to track cognitive changes in non-demented patients with PD (Pagonabarraga et al., 2008; Fernández de Bobadilla et al., 2013). Therefore, PDCRS might be a better tool to detect early MCI compared to MoCA perhaps due to its ability to assess not only fronto-subcortical but also posterior cortical deficits in PD. Its item of alternating verbal fluency and delayed verbal memory independently differentiated the PD-MCI group from both controls and cognitively intact PD patients (Pagonabarraga et al., 2008).

The CTMT was used in our study to particularly assess executive dysfunction. Based on the CTMT, 8 patients (27.6%) with normal cognition on MoCA had executive dysfunction, whereas 17 patients (77.3%) with MCI were found to have severe impairment of executive dysfunction. This implies that MoCA alone is perhaps not adequate to detect executive dysfunction in early PD and may underestimate the severity of executive dysfunction. In support of our findings, previous studies have also shown that the cognitive profile of PDD patients is typically a subcortical dementia syndrome with a greater impairment in the non-amnestic cognitive domains such as executive function, attention and visuospatial function with less impairment in memory, language and praxis (Goldman and Litvan, 2011). Similarly, another study showed that PD patients whom were not known to have dementia do report being distractible and forgetting important information from the previous day more often than healthy controls (Poliakoff and Smith-Spark, 2008).

Our study attempted to explore the association between plasma α-synuclein levels with cognitive impairment in PD patients. Plasma α-synuclein was chosen based on previous studies (Borghi et al., 2000; El-Agnaf et al., 2003) which showed that α-synuclein is released by neurons and is present in extracellular biological fluids like cerebrospinal fluid and plasma. It has been reported that an increased expression of α-synuclein protein alone can result in PD-associated dementia (Singleton et al., 2003). Brain biopsies of PD patients showed that α-synuclein-positive Lewy bodies were associated with cognitive impairment in PD patients independent of AD type of pathology (Mattila et al., 2000). It has been suggested that clinical assessments of plasma α-synuclein concentration may also be of value in identifying groups at risk of developing synucleinopathies, or of having a more aggressive disease course, and for monitoring disease responses to treatments (Fjorback et al., 2007). Our study however, did not demonstrate any association between plasma α-synuclein levels and cognitive impairment in PD patients. Perhaps this is because we did not compare plasma α-synuclein levels with normal controls, as it is well established that plasma α-synuclein is elevated in PD compared to controls (Lin et al., 2017). Lin and colleagues reported a positive association between cognitive decline and plasma α-synucleinin their PD patients compared to normal controls (Lin et al., 2017). Another study conducted among South Korean patients showed that plasma α-synuclein levels were significantly elevated in both PD and MSA patients compared to controls, but did not correlate with age of patients or disease duration (Lee et al., 2006). Their mean α-synuclein level was 79.9 pg/ml whereas our median α-synuclein level was only 18.10 pg/ml. This discrepancy could be attributed to differences in the types of assay or methods used to quantify α-synuclein (Stefanis, 2012). While we acknowledge that plasma α-synuclein may not have been a good biomarker for early cognitive impairment in PD patients, we also did not find any significant differences in the burden of plasma α-synuclein among those with normal cognition and mild cognitive impairment. This could be explained by the fact that plasma α-synuclein is more likely to be a diagnostic biomarker for PD rather than a cognitive biomarker.

Apolipoprotein E genetic polymorphism and *ApoEe4* alleles are known to be associated with Alzheimer's disease (Benmoyal-Segal and Soreq, 2006). Overlapping pathologies between Alzheimer's disease and PD suggest a possible role for *ApoE* in the etiology of PD. Previous studies on *ApoE* genotypes and PD have produced conflicting results (Gao et al., 2011). A meta-analysis by Huang et al. showed that *ApoEe4* allele appeared to be associated with a higher prevalence of dementia in PD patients (Huang et al., 2006). A longitudinal follow up study involving PD patients did not show significant association between *ApoEe4* carrier status and the risk of dementia or rate of cognitive decline despite a higher number of *ApoEe4* carrier status in the PDD compared to non-dementia PD patients (Williams-Gray et al., 2009). Our study findings did not reveal any differences in the *ApoE* polymorphism and the frequency of *e4* allele between the various cognitive subgroups using the MoCA and PDCRS. However, using the CTMT, there was a significant difference in the *ApoEe4* carrier status between patients with executive dysfunction (85.7%) and without executive dysfunction (14.3%). It is unclear why this is so. Perhaps *ApoEe4* is more reflective of executive dysfunction, which is better detected by the CTMT, rather than global cognitive assessment tests such as MoCA and PDCRS, which may underestimate the degree of executive dysfunction. Interestingly, patients with executive dysfunction had a significantly higher frequency of carrying at least one *e4* allele compared to the patients without executive dysfunction. This finding is interesting and is partly supported by a previous prospective study where the authors found that PD patients carrying one or two *ApoEe4* alleles had more severe cognitive impairment, possibly reflecting an increased risk for developing dementia (Engelborghs et al., 2003). This is also consistent with the recent study by Morley et al on the genetic influence on the cognitive decline in PD patients. They found that that carrying at least one *ApoEe4* allele is associated with more rapid cognitive decline in PD and accelerated cognitive decline observed in *ApoE* carriers is not simply the result of an increased risk of coexisting AD, but rather a disease-specific effect of *ApoE* gene status on cognition in PD (Morley et al., 2012).

There were several limitations in this study. As this study was conducted as a pilot study, the small sample size may not reflect the true associations between α-synuclein levels, *ApoE* genotypes and cognitive impairment in PD. Larger studies are needed to further explore this association. Cross sectional design of study limits study findings. It would be better if it was conducted as a prospective longitudinal study with long term follow up, to determine the risk of developing dementia in the PD cohort. Due to lack of controls we were unable to determine if plasma α-synuclein levels were truly elevated in PD and whether there are any differences in *ApoE* genotype carrier frequencies between PD and controls. Additionally, we only established Level 1 MDS criteria for MCI in our PD patients. Ideally, Level 2 criteria would have been preferred to confirm the presence of MCI using more detailed neuropsychological testing.

In conclusion, we discovered that the three neuropsychological tests yielded variable results in detecting cognitive impairment. The PDCRS was somewhat better than MoCA in detecting MCI, while the CTMT, was is specific for executive dysfunction, which is recognized to be one of the earliest features of cognitive impairment in PD. We failed to find an association between plasma α-synuclein levels, and cognitive impairment in our PD patients. However, this result should be verified in larger population, matched with controls. Interestingly, genotype *e3/e4* and being a carrier of e4 allele of the *ApoE* gene correlated with the presence of executive dysfunction in our PD patients. These findings add new perspectives to our understanding of the genetic influence on cognitive impairment in PD, and establish a possible link between *ApoE* and cognitive impairment in PD.

## Author contributions

NS: performed cognitive assessments (MoCA), recruited participants, wrote the first draft, biomarker studies; NA: biomarkers studies, wrote the first draft and substantive editing; MA: biomarkers studies; NM: contributed to the overall design of the study, constructive feedback, recruited participants, obtained funding and provided overall editing and critical review of the manuscript. KM: performed cognitive assessments (PDCRS and CTMT).

### Conflict of interest statement

The authors declare that the research was conducted in the absence of any commercial or financial relationships that could be construed as a potential conflict of interest.
